# Fracaso virológico de la terapia antirretroviral en pacientes *naïve* con HIV: una mirada desde variables farmacológicas

**DOI:** 10.7705/biomedica.7947

**Published:** 2026-08-20

**Authors:** Carlos Alberto Gómez-Mercado, Paulo Andrés Giraldo-Sánchez, Newar Andrés Giraldo-Alzate, Ana María Hincapié-Zapata, Juliana Madrigal-Cadavid, Jorge Iván Estrada-Acevedo

**Affiliations:** 1 Dirección de Generación de Valor, Cielum Health, Medellín, Colombia Dirección de Generación de Valor Cielum Health Medellín Colombia; 2 Dirección de Investigación, Desarrollo e Innovación, helPharma, Medellín, Colombia Dirección de Investigación, Desarrollo e Innovación helPharma Medellín Colombia; 3 Facultad de Ciencias de la Vida y la Salud, Universidad CES, Medellín, Colombiae Universidad CES Facultad de Ciencias de la Vida y la Salud Universidad CES Medellín Colombia

**Keywords:** infecciones por VIH, terapia antirretroviral altamente activa, carga viral, resultado del tratamiento, HIV infections, antiretroviral therapy, highly active, viral load, treatment outcome

## Abstract

**Introducción.:**

La terapia antirretroviral de gran actividad ha demostrado mejorar la supervivencia a largo plazo de los pacientes con HIV. Sin embargo, una proporción considerable de pacientes presenta fracaso o falla virológica durante el tratamiento.

**Objetivo.:**

Determinar la relación entre la polimedicación, el cambio de tratamiento, la falta de adhesión y el no reclamar el medicamento, y la falla virológica de los pacientes con HIV que recibieron tratamiento antirretroviral.

**Materiales y métodos.:**

Se llevó a cabo un estudio observacional de corte transversal con análisis multivariado de personas con HIV en tratamiento antirretroviral atendidos en una institución de salud colombiana entre el 2007 y el 2022, de un censo de 4441 pacientes, en el que se calculó la regresión logística binaria múltiple con fines explicativos y se calcularon los *odds ratios* crudos y ajustados, los intervalos de confianza y el valor de p.

**Resultados.:**

La proporción de falla virológica fue del 28,1 %. En el modelo ajustado se demostró que la polimedicación, el cambio de tratamiento, la falta de adhesión al tratamiento y el no reclamar el medicamento, explicaban de manera significativa la falla virológica.

**Conclusiones.:**

La polimedicación, el cambio de tratamiento, la falta de adhesión al tratamiento y el no reclamar el medicamento, son variables que explican la falla virológica de los pacientes con HIV que recibían terapia antirretroviral.

## Abstract (en)

**Introduction.:**

Highly active antiretroviral therapy has been shown to improve long-term survival in patients with HIV. However, a considerable proportion of patients experience virologic failure during treatment.

**Objective.:**

To determine the relationship between polypharmacy, treatment changes, non-adherence, and failure to claim medication, and virologic failure in HIV patients undergoing antiretroviral therapy.

**Materials and methods.:**

Cross-sectional observational study with multivariate analysis in people with HIV undergoing antiretroviral treatment at a Colombian health institution between 2007-2022, in a census of 4441 patients, where a multiple binary logistic regression was performed with explanatory factors and crude and adjusted OR, confidence intervals and p were calculated.

**Results.:**

The proportion of virologic failure was 28.1%. The adjusted model demonstrated that polypharmacy, treatment changes, non-adherence, and failure to claim medication were significantly associated with virologic failure.

**Conclusion.:**

Polypharmacy, treatment changes, non-adherence, and failure to claim medication explain virologic failure in HIV patients undergoing ART.

La terapia antirretroviral ha transformado el curso de la infección por el HIV, permitiendo mejorar la supervivencia y la calidad de vida de los pacientes infectados. Desde su implementación, varios estudios globales han demostrado una reducción significativa de la morbimortalidad asociada con el HIV, especialmente en los países con acceso oportuno y continuo al tratamiento ^1^^,^^2^. Sin embargo, el fracaso o falla virológica persiste como un desafío crítico, especialmente en las poblaciones con barreras estructurales, socioeconómicas y clínicas. Este fenómeno, definido por la incapacidad de alcanzar o mantener la supresión viral efectiva, compromete los beneficios de la terapia antirretroviral y aumenta el riesgo de falla virológica y la resistencia a los medicamentos.

Diversos estudios han identificado algunos factores que la explican, entre ellos, la polimedicación, la poca adhesión al tratamiento, las interrupciones en reclamar los medicamentos y los cambios de tratamiento ^3^^-^^6^. La polimedicación en las personas con HIV suele estar relacionada con la presencia de comorbilidades crónicas como hipertensión arterial, diabetes mellitus, dislipidemia, enfermedad cardiovascular, trastornos psiquiátricos y enfermedad renal. El manejo farmacológico concomitante de estas condiciones incrementa la complejidad del esquema terapéutico, el riesgo de interacciones medicamentosas y la carga de efectos adversos, factores que pueden comprometer la adhesión y la supresión viral sostenida ^7^. Por su parte, la escasa adhesión a la terapia antirretroviral continúa siendo uno de los factores determinantes más relevantes del fracaso virológico.

En Colombia y en otros países de Latinoamérica, se han implementado programas orientados a fortalecer la adhesión al tratamiento, que incluyen el seguimiento farmacoterapéutico, la educación estructurada del paciente, el acompañamiento interdisciplinario y las estrategias de recordatorio; no obstante, persisten determinantes psicosociales, estigma, trastornos de salud mental y condiciones socioeconómicas adversas que dificultan el cumplimiento óptimo del tratamiento.

Los estudios realizados en diversas regiones del mundo, desde Etiopía ^8^ hasta Europa ^9^, han documentado de manera constante la relación entre el cumplimiento subóptimo del tratamiento y la menor probabilidad de supresión viral.

En Latinoamérica, barreras estructurales adicionales, como la fragmentación del sistema de salud, los retrasos en las autorizaciones administrativas, las dificultades en la dispensación oportuna de los medicamentos, las interrupciones en la continuidad del aseguramiento y las limitaciones geográficas en el acceso a los servicios especializados, pueden afectar el que los pacientes reclamen el tratamiento y favorecer la falla virológica ^10^^,^^11^.

Por otro lado, los cambios en los esquemas de tratamiento, aunque clínicamente necesarios en ciertos casos, pueden reflejar complejidad terapéutica, antecedentes de intolerancia o dificultades en la respuesta virológica, y se han asociado con resultados subóptimos en los estudios de pacientes de Asia y África ^12^^,^^13^.

En este contexto, el presente estudio busca identificar variables farmacológicas que expliquen la falla virológica, con el propósito de generar información que contribuya al diseño de estrategias de intervención clínica y farmacéutica orientadas a reducir el riesgo de resultados adversos y fortalecer la continuidad terapéutica.

## Materiales y métodos

### Tipo de estudio

Se llevó a cabo un estudio observacional de corte transversal con análisis multivariado.

### Población y muestra

La población de estudio incluyó el censo de registro de los pacientes *naïve* con diagnóstico de HIV al inicio de la terapia antirretroviral, que fueron atendidos en una institución de salud ambulatoria entre el 2007 y el 2022. Los registros analizados contenían información sobre la carga viral al inicio del tratamiento y en evaluaciones subsecuentes a los 6 meses. Se excluyeron los pacientes sin información completa sobre la terapia antirretroviral, sin medición de la carga viral y que llevaban menos de 6 meses de tratamiento para evitar los falsos positivos (tiempo suficiente para garantizar disminución de la carga viral).

### Variables

La falla virológica se definió como la carga viral plasmática igual a 50 o más copias por ml, después de seis meses de iniciada la terapia antirretroviral. Este punto de corte corresponde al límite inferior de detección utilizado por los laboratorios clínicos en el contexto local y a lo establecido en la guía nacional de atención farmacéutica para los pacientes con HIV ^14^.

Se reconoce que las recomendaciones internacionales definen la falla virológica confirmada con umbrales mayores. La Organización Mundial de la Salud (OMS) establece 1000 copias o más por ml en dos mediciones consecutivas tras, al menos, seis meses de tratamiento ^15^, mientras que el *Department of Health and Human Services* (DHHS) considera como falla virológica una carga viral confirmada de 200 o más copias por ml ^16^. Los valores ligeramente superiores al límite de detección pueden corresponder a episodios transitorios de viremia de bajo nivel *(blips)* y no necesariamente a fracaso terapéutico confirmado; no obstante, el criterio adoptado en este estudio se aplicó de manera uniforme en toda la población, garantizando coherencia interna para el análisis de las asociaciones.

Además, se recopilaron y analizaron variables sociodemográficas (edad, sexo, estrato socioeconómico, nivel educativo) y farmacológicas (polimedicación, cambio de tratamiento, adhesión al tratamiento y el no reclamar los medicamentos). Estas variables se consideraron en el momento de evaluar el desenlace o al realizar la última medición disponible para cada paciente.

Se consideraron polimedicados a los pacientes que tomaban cinco o más medicamentos de uso crónico -antihipertensivos, hipoglucemiantes, estatinas, psicofármacos, etc.-, comúnmente asociados al manejo de comorbilidades como hipertensión arterial, diabetes mellitus, dislipidemia, trastornos psiquiátricos y enfermedad cardiovascular, entre otros.

El grado de adhesión al tratamiento se determinó utilizando la información recopilada por el farmacéutico durante la consulta de seguimiento farmacoterapéutico; en este proceso, se aplicaron dos herramientas. La primera fue la prueba de Morisky-Green, herramienta ampliamente utilizada en clínica e investigación para medir la adhesión terapéutica, compuesta por cuatro preguntas dicotómicas (sí o no) que detectan barreras para el cumplimiento. Esta herramienta también se conoce como MAQ o MMAS-4, y ha sido validada en enfermedades crónicas como hipertensión, diabetes y HIV ^17^.

La segunda herramienta usada fue la escala SMAQ *(Simplified Medication Adherence Questionnaire),* un cuestionario validado en una amplia cohorte de pacientes españoles para medir la adhesión a la terapia antirretroviral. Esta escala está basada en la prueba de Morisky-Green, pero con modificaciones: se eliminó la pregunta 3 por irrelevancia en los pacientes asintomáticos, se reformuló la pregunta 4 y se añadieron tres preguntas para mayor especificidad.

Se clasifica como «no adherente» a quienes responden de manera negativa o inadecuada a las preguntas cualitativas clave, o según las opciones C, D o E en la pregunta 5 ^18^. Ambas mediciones se combinaron, considerando a un paciente como «adherente» únicamente cuando sus respuestas fueron coherentes en ambos instrumentos.

La tasa de reclamar los medicamentos en farmacia se evaluó a partir de los registros mensuales de dispensación del servicio farmacéutico; este indicador se calculó como el porcentaje de dispensaciones efectivamente hechas en relación con el número total de dispensaciones esperadas según la duración del tratamiento del paciente y un registro inferior al 80 % se consideró como bajo.

### Recolección de la información

La información se obtuvo a partir de la revisión de los registros clínicos y las bases de datos institucionales correspondientes al periodo 2007-2022. Se extrajeron las variables sociodemográficas, farmacológicas y virológicas mediante un instrumento estructurado en Microsoft Excel™ y diseñado para el estudio.

La base de datos fue depurada y validada antes del análisis, garantizando coherencia y completitud de la información. El sesgo de selección se controló incluyendo el 100 % de los registros disponibles y los sesgos de información mediante la aplicación de criterios de exclusión.

### Análisis estadístico

El análisis de los datos se llevó a cabo mediante procedimientos univariados, bivariados y multivariados, utilizando R Studio *(software* de libre acceso). Inicialmente, se calcularon las medidas de frecuencia para las variables cualitativas, mientras que. para la variable dependiente, se evaluó la normalidad mediante la prueba de Kolmogorov-Smirnov; se evidenció que la carga viral presentó una distribución asimétrica, razón por la cual se decidió reportarla con la mediana y el rango intercuartílico, en lugar de la media y la desviación estándar. Además, se generaron medidas de resumen para las demás variables.

En el análisis bivariado, se aplicó la prueba de ji al cuadrado y se hizo una regresión logística binaria simple para calcular los *odds ratios* crudos junto con sus intervalos de confianza del 95 %. Finalmente, se desarrolló un modelo de regresión logística binaria múltiple con fines explicativos, para controlar la confusión y obtener los *odds ratios* ajustados.

### Consideraciones éticas

Esta investigación contó con el aval del Comité Institucional de Ética para Investigación en Humanos de la Universidad CES, y con la aprobación de la Dirección de Investigaciones de *helPharma.* De acuerdo con el artículo 11 de la Resolución 008430 de 1993 del Ministerio de Salud de Colombia, el estudio fue clasificado como investigación «sin riesgo», dado que se basó en la revisión retrospectiva de información previamente existente en bases de datos institucionales, sin realizar intervenciones directas sobre los pacientes.

Se incluyeron registros de pacientes adultos y algunos menores de edad en tratamiento antirretroviral.

El Comité de Ética consideró que, al tratarse del análisis secundario de datos clínicos previamente recolectados y completamente anonimizados, no se requería consentimiento informado individual. Se garantizó en todo momento la confidencialidad y privacidad de la información mediante el anonimato en los datos de identificación personal y el acceso restringido a la base de datos.

## Resultados

Se incluyeron 4438 pacientes con HIV que estaban recibiendo terapia antirretroviral. La carga viral, medida por las copias de virus en sangre, presentó una distribución asimétrica (p < 0,01). La mediana fue de 40 copias/ml (RIC: 28 a 64), con valores mínimos de 16 y máximos de 961 371 copias/ml. Respecto a la estimación de la proporción de prevalencia de falla virológica, se halló que el 28,1 % de los pacientes había presentado falla virológica (el 22,1 % entre 50 y 99 copias/ml y el 6 % 1000 copias/ml), mientras que la proporción restante obtuvo éxito terapéutico.

Entre las variables demográficas, se halló que la media de la edad de los pacientes fue de 41,5 (DE = 12,7). La edad mínima fue de 3 años y la máxima de 98 años. El 89,3 % eran hombres. El 78,3 % residía en viviendas de estrato socioeconómico bajo, o igual o menor de 3; es decir que pertenecían a un grupo socioeconómico con menores recursos. El 78,8 % tenía un nivel educativo profesional o con posgrado. En el análisis bivariado se halló una relación no significativa entre las variables demográficas (sexo, estrato y nivel educativo) y el resultado.

En cuanto a las variables farmacológicas, se evidenció que el 66,2 % estaba polimedicados, el 7,7 % requirió cambio de tratamiento, el 11,5 % no había tenido adhesión a la terapia antirretroviral y el 10,5 %, en algún momento, había dejado de reclamar el medicamento en menos del 80 % de las oportunidades (cuadro 1).

**Cuadro 1:** Características demográficas y farmacológicas de los pacientes con HIV que recibieron terapia antirretroviral entre el 2017 y el 2022

Variable	Categoría	n	%
Sexo	Mujer	473	10,7
	Hombre	3965	89,3
Estrato socioeconómico	Medio o alto (> 3)	963	21,7
	Bajo (≤ 3)	3475	78,3
Nivel educativo	Profesional	3497	78,8
	Tecnólogo	941	21,2
Polimedicado	No	1500	33,8
	Sí	2938	66,2
Cambio de tratamiento	No	4095	92,3
	Sí	343	7,7
Poca adhesión terapéutica	No	3927	88,5
	Sí	511	11,5
El no reclamar el medicamento	No	3970	89,5
	Sí	468	10,5

El 88,8 % de los pacientes polimedicados presentó falla virológica, en comparación con los no polimedicados, y estas diferencias fueron estadísticamente significativas; el tener esta condición aumentó la probabilidad de un resultado desfavorable del desenlace en 5,86 veces (IC_95%_: 4,86-7,10).

También, se evidenció que el presentar poca adhesión terapéutica y el no reclamar los medicamentos eran condiciones que aumentaban las probabilidades de falla virológica. Por otro lado, en aquellos pacientes que tuvieron cambio de tratamiento, las probabilidades de fracaso se redujeron en el 84 % (cuadro 2).

**Cuadro 2:** Relación entre las características demográficas y las farmacológicos con el fracaso virológico en pacientes con HIV que recibieron terapia antirretroviral entre el 2017 y el 2022

Variable	Categoría	Fracaso	Éxito	X^2^	p	OR_c_	IC95%	
		(N = 1245)	(N = 3,193)				LI	LS
		n (%)	n (%)					
Sexo	Mujer	136 (10,9)	337 (10,6)	0,09	0,76	1	-	-
	Hombre	1,109 (89,1)	2,856 (89,4)			0,96	0,78	1,19
Estrato	Medio o alto (> 3)	262 (21,0)	701 (22,0)	0,38	0,53	1	-	-
	Bajo (≤ 3)	983 (79,0)	2,492 (78,0)			1,06	0,90	1,24
Nivel educativo	Profesional	976 (78,4)	2,521 (79,0)	0,13	0,71	1	-	-
	Tecnólogo	269 (21,6)	672 (21,0)			1,03	0,88	1,21
Polimedicación	No	140 (11,2)	1,360 (42,6)	392,00	<0,01	1	-	-
	Sí	1,105 (88,8)	1,833 (57,4)			5,86	4,86	7,10
Cambio de tratamiento	No	1,223 (98,2)	2,872 (89,9)	85,08	<0,01	1	-	-
	Sí	22 (1,8)	321 (10,1)			0,16	0,10	0,24
Poca adhesión terapéutica	No	1,011 (81,2)	2,916 (91,3)	89,04	<0,01	1	-	-
	Sí	234 (18,8)	277 (8,7)			2,44	2,02	2,94
El no reclamar el medicamento	No	1,040 (83,5)	2,930 (91,8)	63,43	<0,01	1	-	-
	Sí	205 (16,5)	263 (8,2)			2,20	1,80	2,67

OR_c_: *odds ratio* crudo; LI: límite inferior; LS: límite superior

En la figura 1 se presenta la comparación entre el modelo crudo y el modelo ajustado, en la que se observa que los pacientes polimedicados presentaron una probabilidad de 5,44 de falla virológica (OR ajustado = 5,44; IC_95%_: 4,50 a 6,63; p < 0,01). Al aplicar el criterio de cambio en el estimador *(change-in-estimate)*^19^, la variación entre el OR crudo y el ajustado fue del 7 %, lo que indica ausencia de factores de confusión relevantes tras el ajuste por edad, sexo, estrato socioeconómico, nivel educativo, cambio de tratamiento, poca adhesión terapéutica y porcentajes de no reclamar los medicamentos. La magnitud del efecto se mantuvo elevada y en la misma dirección, lo que sugiere estabilidad de la asociación.

**Figura 1:**
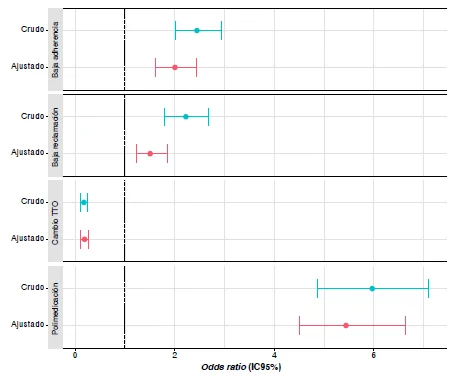
Variables farmacológicas que explican el fracaso virológico en pacientes con HIV que recibieron terapia antirretroviral entre el 2017 y el 2022

De manera constante, la baja adhesión al tratamiento incrementó en el 98 % las probabilidades de fracaso o falla virológica (OR ajustado = 1,98); el no reclamar los medicamentos se asoció con un aumento del 50 % (OR ajustado = 1,50). En estos casos, el cambio porcentual en el estimador fue del 19 y del 32 %, respectivamente, lo que indica la presencia moderada de factores de confusión tras el ajuste; sin embargo, la dirección del efecto se mantuvo constante y fue clínicamente relevante.

Por otra parte, el cambio de tratamiento presentó una asociación inversa con el resultado, çon una reducción del 83 % en las probabilidaes de fracaso virológico (OR ajustado = 0,17). La variación entre el estimador crudo y el ajustado fue del 6 %, lo que sugiere ausencia relevante de factores de confusión y estabilidad del efecto protector observado.

## Discusión

La proporción del fracaso virológico en este estudio fue del 28,1 %, hallazgo que supera lo reportado en las investigaciones de Rusconi *et al.* en Italia (8,4 %) ^8^, Salvana *et al.* en Filipinas (10,3 %) ^20^ y Tarrago *et al.* en Europa (9,8 %) ^21^, pero menor al observado por Young *et al.* en Suiza (35 %) ^22^ y Zhao *et al.* en China (35 %) ^13^.

En el contexto colombiano, los datos nacionales más recientes de la Cuenta de Alto Costo ^23^ indican que, de 185 954 personas registradas con HIV, el 80,7 % recibió terapia antirretroviral y el 89,4 % alcanzó supresión viral (≤ 1000 copias/ml), lo que implica una proporción estimada de falla virológica cercana al 10,6 % a nivel nacional. Sin embargo, estudios más específicos han reportado tasas más elevadas, entre el 9,2 y el 20, 9 %, dependiendo de la región.

En la región Caribe, De La Hoz *et al.*^24^ documentaron una falla virológica del 20,9 % en pacientes que recibían terapia antirretroviral, asociada principalmente a poca adhesión al tratamiento. De manera similar, Gómez *et al.*^4^ reportaron una proporción de fracaso virológico del 16,7 % en una institución colombiana. Estas diferencias sugieren que, aunque Colombia presenta una alta tasa global de supresión viral, persisten subgrupos clínicos con mayor riesgo de fracaso, especialmente cuando confluyen barreras estructurales y farmacológicas. Estas diferencias reflejan que las características demográficas, las condiciones socioeconómicas y las barreras en el acceso a los servicios de salud, influyen en los resultados de la terapia antirretroviral.

En este estudio, la polimedicación se identificó como el principal factor asociado con el fracaso virológico, con un impacto significativo en los resultados terapéuticos. El 66,2 % de los pacientes incluidos registraba polimedicación, lo que aumenta la complejidad del manejo farmacológico y dificulta el cumplimiento de la terapia antirretroviral. Este hallazgo concuerda con el estudio de Ahn *et al.*^3^, en el que se reporta que los pacientes con múltiples comorbilidades y tratamientos simultáneos, enfrentan mayores dificultades para cumplir con los esquemas terapéuticos. La polimedicación, no solo complica el cumplimiento, sino que también puede incrementar los efectos adversos y reducir la efectividad de la terapia antirretroviral, subrayando la necesidad de optimizar y simplificar los esquemas farmacológicos en esta población.

En las personas con HIV, la transición epidemiológica hacia una población más envejecida ha incrementado la carga de comorbilidades crónicas no transmisibles. Los estudios internacionales han documentado una gran prevalencia de hipertensión arterial, diabetes mellitus de tipo 2, dislipidemia y enfermedades cardiovasculares, así como trastornos depresivos y ansiedad, condiciones que requieren tratamiento con antihipertensivos, hipoglucemiantes, estatinas, antidepresivos y benzodiacepinas ^25^. La coexistencia de estos medicamentos con los esquemas antirretrovirales puede generar interacciones farmacocinéticas y farmacodinámicas, especialmente con los inhibidores de proteasas y con algunos inhibidores no nucleósidos de la transcriptasa inversa, lo cual afecta las concentraciones plasmáticas y la tolerabilidad ^26^.

En investigaciones previas de poblaciones con HIV, se ha señalado que la polimedicación se asocia con mayores riesgos de eventos adversos, menor adhesión al tratamiento y potencial incremento de las probabilidades de falla virológica, especialmente en los pacientes con multimorbilidades ^27^.

Uno de los factores determinantes de la falla virológica identificada en este estudio fue la poca adhesión a la terapia antirretroviral. Este hallazgo concuerda con lo reportado en estudios previos, como el Glass *et al.*^28^, el cual evidencia que la falta de adhesión es un factor significativo del fracaso virológico y de la mortalidad. La relación entre la poca adhesión al tratamiento y la falla virológica refuerza la necesidad de implementar intervenciones que promuevan el seguimiento y la adhesión a los tratamientos.

En Colombia, el modelo de atención integral en HIV ha incorporado estrategias de seguimiento farmacoterapéutico, la entrega programada de los medicamentos, la educación al paciente y el monitoreo periódico de la carga viral, lo que ha permitido alcanzar niveles de supresión viral cercanos al 89 %, según los datos nacionales recientes ^23^. Algunas instituciones prestadoras de servicios de salud han implementado modelos de dispensación continua y seguimiento interdisciplinario, que han demostrado efectividad en la retención en el cuidado y continuidad del tratamiento, aun en contextos de crisis estructural del sistema sanitario.

La gestión farmacéutica enfrenta retos significativos, como los recursos limitados, las barreras económicas, la falta de acceso oportuno a los medicamentos y las limitaciones en los sistemas de salud, situaciones que restringen la posibilidad de mantener la adhesión terapéutica y que tienen relación con el fracaso virológico en pacientes con HIV, como lo han demostrado Bircher *et al.* en Tanzania ^29^ y Nanfuka *et al.* en Uganda ^30^.

Considerando lo anterior, una solución eficaz para identificar y apoyar a los pacientes en riesgo es el uso de las herramientas predictivas y de las tecnologías para monitorear la adhesión en tiempo real, como las empleadas por Woodward *et al.*^31^ y Benítez *et al.*^32^, que permiten intervenciones personalizadas para optimizar los resultados clínicos, reduciendo las probabilidades de fracaso virológico.

Otro factor relevante fue el no reclamar los medicamentos, presente en el 10,5 % de los pacientes. Este indicador refleja problemas en la continuidad del acceso al tratamiento, un aspecto fundamental para garantizar la eficacia de la terapia antirretroviral. En un estudio previo en Colombia, se encontró que las interrupciones en la dispensación, ya sea por barreras administrativas, desfase en las autorizaciones o problemas económicos, se asociaban directamente con el fracaso virológico ^33^.

Por último, el cambio de tratamiento, presente en el 7,7 % de los casos, también se asoció con el fracaso virológico, aunque en menor medida que las variables previamente mencionadas. Este hallazgo concuerda con lo reportado por Mantshonyane *et al.*^33^, quienes demostraron que los cambios en el esquema terapéutico -muchas veces derivados de reacciones adversas o problemas de adhesión- tienden a reducir la efectividad de la terapia antirretroviral y a aumentar el riesgo de fracaso. Aunque el cambio de tratamiento puede ser inevitable en ciertos casos, es crucial que estas decisiones se hagan bajo un seguimiento estrecho y en contextos en los cuales se garantice la adhesión al nuevo esquema terapéutico.

Una fortaleza relevante de este estudio radica en la inclusión del censo completo de los registros entre el 2017 y el 2022, excluyendo únicamente a los pacientes con menos de seis meses de tratamiento antirretroviral. Esta estrategia permitió minimizar el sesgo de selección y fortalecer la representatividad de los resultados dentro del contexto institucional analizado. No obstante, el diseño transversal limita la posibilidad de establecer relaciones causales, y el uso de información secundaria restringió la incorporación de variables potencialmente confusas, particularmente aquellas de naturaleza social y psicológica, que podrían aportar una comprensión más amplia del fenómeno.

Además, aunque la adhesión terapéutica fue evaluada mediante instrumentos validados de autorreporte, este tipo de medición no registra de manera integral los factores determinantes psicosociales, culturales y estructurales que influyen en la continuidad terapéutica. Factores como el estigma, el apoyo social, la percepción de enfermedad, las experiencias previas con el sistema de salud y las dinámicas familiares pueden desempeñar un papel sustancial en los resultados virológicos. En este orden de ideas, investigaciones futuras con enfoques cualitativos o diseños mixtos podrían complementar los hallazgos cuantitativos y ofrecer una visión más integral del fracaso virológico en personas con HIV.

Por otra parte, la definición operativa de fracaso virológico utilizada en este estudio (≥ 50 copias/ml) corresponde al límite inferior de detección del laboratorio y puede diferir de los criterios clínicos confirmatorios establecidos por la OMS (≥ 1000 copias/ml) y el DHHS (≥ 200 copias/ml). La adopción de puntos de corte más elevados podría modificar la frecuencia observada del resultado y, en consecuencia, su interpretación clínica. Sin embargo, el criterio seleccionado permitió analizar asociaciones farmacológicas bajo un enfoque sensible a la capacidad de detección viral en el contexto local. Los futuros estudios podrían incorporar un análisis de sensibilidad empleando diferentes puntos de corte.

Finalmente, se reconoce que el cambio de terapia antirretroviral podría estar influenciado por episodios previos de fracaso virológico o por dificultades en la adhesión al tratamiento, lo que introduce la posibilidad de causalidad inversa o confusión residual.

En conclusión, la polimedicación, la poca adhesión al tratamiento, el bajo porcentaje de reclamar los medicamentos y el cambio de tratamiento son factores que explican el fracaso virológico en esta población. Estos hallazgos subrayan la necesidad de optimizar los esquemas terapéuticos, garantizar la continuidad del acceso a los medicamentos y diseñar estrategias de apoyo integral que aborden tanto las barreras individuales como las estructurales. La implementación de modelos de manejo multidimensional y de herramientas tecnológicas de monitoreo, puede contribuir a mejorar los resultados clínicos y garantizar la sostenibilidad de la terapia antirretroviral en las poblaciones vulnerables.
